# Information systems for mental health in six low and middle income countries: cross country situation analysis

**DOI:** 10.1186/s13033-016-0094-2

**Published:** 2016-09-26

**Authors:** Nawaraj Upadhaya, Mark J. D. Jordans, Jibril Abdulmalik, Shalini Ahuja, Atalay Alem, Charlotte Hanlon, Fred Kigozi, Dorothy Kizza, Crick Lund, Maya Semrau, Rahul Shidhaye, Graham Thornicroft, Ivan H. Komproe, Oye Gureje

**Affiliations:** 1 Transcultural Psychosocial Organization Nepal, Kathmandu, Nepal; 2 Department of Research and Development, HealthNet TPO, Amsterdam, The Netherlands; 3 Institute of Psychiatry, Psychology and Neuroscience, Centre for Global Mental Health, King’s College London, London, UK; 4 Department of Psychiatry, College of Medicine, University of Ibadan, Ibadan, Nigeria; 5 Centre for Chronic Conditions and Injuries, Public Health Foundation of India, New Delhi, India; 6 Department of Psychiatry, School of Medicine, College of Health Sciences, Addis Ababa University, Addis Ababa, Ethiopia; 7 Butabika National Mental Hospital, Kampala, Uganda; 8 Department of Psychiatry and Mental Health, Alan J Flisher Centre for Public Mental Health, University of Cape Town, Cape Town, South Africa; 9 Health Service and Population Research Department, Institute of Psychiatry, Psychology and Neuroscience, King’s College London, London, UK; 10 Utrecht University, Utrecht, The Netherlands; 11 CAPHRI School for Public Health and Primary Care, Maastricht University, Maastricht, Netherlands

**Keywords:** Mental health, Information systems, Low and middle income countries

## Abstract

**Background:**

Research on information systems for mental health in low and middle income countries (LMICs) is scarce. As a result, there is a lack of reliable information on mental health service needs, treatment coverage and the quality of services provided.

**Methods:**

With the aim of informing the development and implementation of a mental health information sub-system that includes reliable and measurable indicators on mental health within the Health Management Information Systems (HMIS), a cross-country situation analysis of HMIS was conducted in six LMICs (Ethiopia, India, Nepal, Nigeria, South Africa and Uganda), participating in the ‘Emerging mental health systems in low and middle income countries’ (Emerald) research programme. A situation analysis tool was developed to obtain and chart information from documents in the public domain. In circumstances when information was inadequate, key government officials were contacted to verify the data collected. In this paper we compare the baseline policy context, human resources situation as well as the processes and mechanisms of collecting, verifying, reporting and disseminating mental health related HMIS data.

**Results:**

The findings suggest that countries face substantial policy, human resource and health governance challenges for mental health HMIS, many of which are common across sites. In particular, the specific policies and plans for the governance and implementation of mental health data collection, reporting and dissemination are absent. Across sites there is inadequate infrastructure, few HMIS experts, and inadequate technical support and supervision to junior staff, particularly in the area of mental health. Nonetheless there are also strengths in existing HMIS where a few mental health morbidity, mortality, and system level indicators are collected and reported.

**Conclusions:**

Our study indicates the need for greater technical and resources input to strengthen routine HMIS and develop standardized HMIS indicators for mental health, focusing in particular on indicators of coverage and quality to facilitate the implementation of the WHO mental health action plan 2013–2020.

## Background

The health management information system (HMIS) is an integral part of all health systems because it aims to provide reliable and timely information on treatment needs and resource demands on the health system [1]. Sauerborn and Lippeveld [2] defined such a system as “a set of components and procedures, organized with the objectives of generating information, which will improve health care management decisions at all levels of the health system”. The HMIS is a component or sub-system of the Health Information System (HIS) that refers to the health services data collected at a facility level [3]. Within the broader six components of HIS; resources, indicators, data sources, data management, information products and dissemination and use [4], the HMIS specifically looks at medical records of hospitals or health care organizations and deals largely with the accumulation, storage and accuracy of patient or individual related data. In the long term, HMIS has the potential to improve governance, transparency, accountability, evidence-based decision making, quality of services and performance-based financing strategies that are geared towards meeting the needs of the population [5]. In the short term, HMIS is an important tool for the planning and management of health services [6], as well as resource prioritisation.

HMIS is of universal importance, particularly in low and middle-income countries (LMICs) that are characterized by poor health outcomes, human resource shortages and limited financial resources. The reality is that the health systems, including the health information systems, of LMICs are often quite weak and fragmented, such that they fail to meet the needs of service providers and policy makers [7]. In several countries, for example, a large volume of routinely collected HMIS data eventually reaches the national level without being cross-checked, analyzed or utilized [2].

Despite increasing attention and investment in recent years, HMIS in LMICs face challenges of poor data quality, lack of qualified human resources, low management capacity, inadequate infrastructure, insufficient space for HMIS and technological difficulties such as software malfunctioning, data loss due to computer viruses or irregular electricity power supply [8]. Therefore, despite its potential to be a strong health system strengthening tool, the benefits of HMIS remain largely unrealized [9].

To make the best use of HMIS for mental health system strengthening, it is important to review the existing state of affairs of mental health within HMIS. Firstly, because there is an urgent need to develop mental health services in LMICs based on the high global burden of disease attributed to mental, neurological, substance abuse (MNS) disorders and self-harm (12 %) [10]; and implement the WHO mental health action plan [11] and Mental Health Gap Action Program (mhGAP) guidelines for the integration of mental health into primary health care [12].

Secondly, the restructuring of existing HMIS has become a necessity, in a situation where primary health care has become a global priority [13] and funding for health is accompanied by greater demand for reliable statistics to track progress [14]. Thirdly, the absence of reliable data collection to accurately capture the mental health situation within the HMIS limits the capacity of mental health professionals and advocates to lobby for more investments to address the huge burden of mental disorders. It also hinders evidence-based improvements in the organization and provision of mental health care services to address specific areas of priority needs. This is because meaningful planning and projections cannot be carried out without reliable data.

The literature regarding the development of HMIS is scarce [2] and even more so for “mental health component within HMIS”, as there is a lack of health care system focus on mental health.

The ‘Emerging mental health systems in low and middle income countries’ (Emerald) research programme aims to support mental health systems strengthening in the six countries of Ethiopia, India, Nepal, Nigeria, South Africa and Uganda [15] a key area of focus of the Emerald program is to strengthen the mental health component of the HMIS in the participating countries, through development and field testing of suitable mental health indicators to monitor the performance of the mental health system. A necessary preliminary step is to perform a situation analysis of the current state of HMIS, and the mental health components within HMIS, in all the participating countries to map the current situation and identify the gaps. This paper presents a situation analysis to highlight the strengths, challenges and opportunities for developing and strengthening “mental health components” within routine government HMIS across the participating countries.

## Methods

### Setting

See Table 1 for details of the Emerald country sites. The Emerald countries represent two continents (Africa and Asia) and have three income levels (upper middle-income: South Africa; lower middle-income: India and Nigeria; and low-income, Ethiopia, Uganda and Nepal) and include a fragile state (Nepal).

**Table 1:** Socio-demographic characteristics of Emerald countries

	Ethiopia	India	Nepal	Nigeria	South Africa	Uganda
Population (in millions)^a^	95.9	1296.2	27.1	177.5	53.7	38.8
Proportion of population living on <$1.25 per day^b^	30.65	32.68	24.82	67.98	13.77	38.01
Human development index rank^b^	173	135	145	152	118	164
Population growth rate^a^	2.1	1.5	1.5	2.5	1.0	3.4
Maternal mortality rate^b^	350	200	170	630	300	310
Infant mortality rate^a^	50	44	46	69	42	57
Gross domestic product per capita (USD)^b^	1218	5050	2131	5440	11,989	1334
% Gross domestic product spent on health^b^	4.7	3.9	5.4	5.3	8.5	9.5
% health budget spent on mental health^c^	0.07	2.05	0.08	0.40	4.50	0.44

^a^Population Reference Bureau [28]

^b^UNDP [29]

^c^WHO [30]

### Broader country contexts for health information management

The HMIS, which aims to assist in the management and planning of health programmes, has diverse history in Emerald countries. For example, in Uganda the HMIS was introduced in 1997, to improve the pre-existing health information system introduced in 1985 [16]. In South Africa HMIS was established after 1994, as during the apartheid system, health services were extremely fragmented, and there were inequitable health data standards. In 2001, South Africa was able to establish national standards, with essential data and indicator sets, which all provinces are required to report [17]. In India, as part of the National Health Rural Mission that began in 2005, the HMIS received greater emphasis and was expected to improve governance as well as the monitoring of the health system [18]. In 1991 the national health policy in Nepal recognized the need for health sector information and since then, there have been several initiatives, the latest being the revision of existing HMIS indicators to meet current needs [19]. In Ethiopia, the reformed HMIS was pilot tested in 2006/07and since September 2009, the HMIS scale-up project has provided training to health workers of the Southern Nations and Nationalities People’s Region [20]. Nigeria had a weak health system when it became independent in 1960. It began health reforms after the Alma Ata conference of 1978, but required 10 years to establish the national health policy in 1988 which contained provisions for a robust country HMIS [21].

### Study design

We conducted a cross-country situation analysis to obtain information from key documents in the public domain, and supplemented this by contact with key officials in government services where necessary.

### Instrument

The instrument was developed by three of the authors (NU, MJ, OG) and was revised after inputs from consortium partners. The situation analysis tool (http://www.emerald-project.eu/tools-instruments/) had nine sections which covered background of the HMIS, plans and policies related to HMIS, the process of recording and collating data, monitoring, evaluation and feedback procedures, dissemination and utilisation of data, human resources, availability of mental health indicators, coordination and linkages, and an open section for any other relevant issues not covered in the previous sections.

### Data collection

The coordinators and research staff in each of the study sites completed the situation analysis by reviewing secondary documents and engaging in informal interviews with government HMIS staff between March and May 2013. All the sources utilised to answer the situation analysis tool were documented and were updated as and when new information was available. The country teams reviewed the completed in-country data for comprehensiveness and comprehensibility. In cases where inconsistencies were noted, further cross-checking of the collected information was performed.

### Data analysis

The data from all six countries were collated and tabulated in an Excel spreadsheet, based on the nine sections outlined above. The responses for each question were coded and summarized. During this process two researchers checked if all the questions were answered properly and if responses were understandable. The aspects that required further clarifications were noted. The coding and summarization process meant that similar information was grouped into one category or theme, and for each theme a summary table was developed. Preliminary results were sent to all country partners with requests for additional information, clarifications and for a validation check of the findings. Further feedback and information was incorporated to derive the final results.

## Results

### Policy context for mental health and HMIS

All six countries have an operational HMIS that is overseen by the respective departments or directorates under the Ministry of Health. None of the countries possess a separate policy for mental health information management, but the mental health policies in some countries (Nepal, South Africa and Uganda) and the mental health strategy in Ethiopia have sections on mental health data collection and management (see Table 2).

**Table 2:** Policies and plans related to mental health and HMIS

Themes	Ethiopia	India	Nepal	Nigeria	South Africa	Uganda
Mental health policy or plan	Yes^a^	Yes^b^	Yes	Yes	Policy drafted	Yes
Provisions for MHIS^c^ in mental health policy	Yes, but not implemented	Yes	Yes. Plan to maintain record systems	No	Yes. A section on MHIS	Yes
Provisions in mental health policy for social welfare benefits	No	Yes, talks about monetary and tax benefits	No	No	Covered in other health policies	No
General health policies that govern HMIS	5-yearly health plan	National Health Policy 2002 and Draft National Health Policy 2015	National Health Policy and Nepal Health Sector Programme-2	Revised Policy Programme and Strategic Plan	White Paper for the Health System District HMIS policy	Health Policy
General health plans that govern HMIS	Health Sector Development Plan IV (2010/11–2014/2015)	The National Rural Health Mission (2005–2012)	Second Long Term Health Plan1997–2017	National 3-5 year Health Plan	5-year health plan	Health Sector Strategic Plan2010/11–2014/15
Standard operating procedures for mental health	No	No	No	No	Yes	No
Initiatives to develop MHIS	Yes	No	No	No	Yes	No

^a^Ethiopia has a National Mental Health Strategy which is the equivalent of a policy/plan

^b^When the data for this study was collected between March–May, 2013, there was no mental health policy in India. It was only in October 2014, India released its first mental health policy in which monitoring and evaluation of national mental health programme has been emphasised

^c^Mental Health Information System

Ethiopia and South Africa, realizing the need for quality data on mental health, have taken policy level advocacy initiatives to integrate mental health indicators within routine HMIS, rather than having a parallel system for mental health information management. As a result, the South African standard operating procedures (SOPs) include processes on how to collect, record and report mental health data in HMIS.

Apart from South Africa, none of the countries have specific HMIS policies for general health conditions, although health policies and plans of those countries mention HMIS guidelines and standard operating procedures (SOPs) which could be helpful in the development of mental health components within HMIS. For example, the 3–5 year health plans in Uganda, South Africa, Ethiopia and Nigeria have laid out plans to implement HMIS. In Nepal, the second long term health plan (1997–2017) and health sector strategy (2002) have emphasized the need for the establishment of a health sector information system (HSIS). In Ethiopia, high-level goals for strengthening HMIS are specified in the ‘National Health Policy of the Transitional Government of Ethiopia, 1993’. The Health sector development plan IV, 2010/2011–2014/2015, includes a commitment to electronic HMIS and specifies the indicators to be measured routinely. In Nigeria, each state Ministry of Health implements the national HMIS plan.

### Situation of HMIS human resources

Table 3 shows human resources for HMIS in the six Emerald countries. Limited human resources are involved in HMIS and staff are mostly junior data entry clerks. In all six countries, there is a small pool of HMIS experts. The pre-service training of HMIS human resources is limited to a few lectures within post-graduate courses of medicine and public health, except in Uganda and Ethiopia which have specialized university courses. In Uganda course in Public Health Informatics is being delivered through the School of Public Health at Makerere University; a diploma course in medical records and HMIS is also offered by the Uganda Institute of Allied Health and Management Sciences. In Ethiopia, there are three universities that offer bachelors (Gondar University) and masters (Addis Ababa, Gondar and Mekelle University) degree courses in health informatics. Apart from these universities there are over seven health information technician (HIT) training regional colleges which have so far trained 2488 people on health informatics. The qualification required to become an HMIS specialist varies across countries, but in all countries previous work experience in HMIS field is required. Nigeria requires a minimum qualification of a Bachelor’s degree in Statistics or Health Management while Nepal and India require a Master’s degree in Statistics or Information Technology. In South Africa, there are no HMIS specialists, because health information management is considered interdisciplinary, so staff from the respective departments manage the health information. India, Nepal and Uganda have twenty, five and three specialists respectively; the remaining countries have no information on the number of specialist staff. Nepal and Nigeria have 200 HMIS trainers each while Uganda has a pool of 10 national trainers. There is no information available for the number of HMIS trainers in Ethiopia and India.

**Table 3:** HMIS human resources

Themes	Ethiopia	India	Nepal	Nigeria	South Africa	Uganda
Minimum qualification needed to start career in HMIS.	Level IV diploma	Graduate in any discipline	Diploma in statistics	Bachelor	This varies widely	Certificate in HIMS
Qualification for HMIS expert	Information not available	BSc and MSc in statistics	MA in Statistics and work experiences	BSc in Health information and work experience	None as the expertise is interdisciplinary	Master degree in biostatistics
Number of HMIS specialists	Number not available	20	5	Number not available	Number not available	3
Number of HMIS trainers	Number not available	Number not available	About 200	About 200	Number not available	About 10
Standard HMIS training manuals	Yes	Yes	Yes	Yes	Yes	Yes
Specialized courses in HMIS	Yes	No	No	Yes, but very few	No	Yes

Across sites, in-service training was given by the HMIS department, though on an ad-hoc basis. All countries had HMIS training manuals, which were widely used in India, Nepal and Uganda. Most of the countries had dedicated HMIS staff at a central and regional level. None of the countries had such staff at the primary health care level but rather utilized other cadres of health care staff such as nurses, auxiliary health workers, community health workers and health assistants to collect and manage the data.

### Mental health indicators collected from routine HMIS

Despite the existence of mental health services, a very limited range of mental health indicators are collected as part of routine HMIS in Ethiopia, Nepal, South Africa and Uganda. In general, the countries tended to collect routine data on health service contacts of people with mental health problems (disease categories) rather than mental health system information such as number of health workers trained in mental health, number of beds available for mental health, the admission rate and number of people being supported from social security funds, as summarised in Table 4. The indicators presented in Table 4 are those reported in HMIS related documents of the six countries.

**Table 4:** Mental health indicators in HMIS

Themes	Ethiopia	India	Nepal	Nigeria	South Africa	Uganda
Mental health indicators in national HMIS	Yes	No^a^	Yes	No^b^	Yes	Yes
Mental health out-patient department (OPD) attendances included	Yes	No	Yes	No	No	Yes
Mental health referrals recorded	No	No	No	No	No	Yes
Psychiatric in-patient bed occupancy rate	No	No	Yes	No	Yes	Yes
Mental health training data reflected	No	No	Yes	No	No	No
Average length of stay at the hospital	No	No	No	No	Yes	Yes

^a^However, State level mental health programme have guidelines for reporting data on MH in states like madhya Pradesh. Data on admission in tertiary level mental hospital and days spent in mental hospital are recorded

^b^However, data on mental health outpatient visit, patients treated at day care facilities, psychiatric bed of general hospitals and mental hospital are collected

We found that all countries record some mental health indicators, but that these countries vary in their categorization of mental health problems. In Ethiopia, data on five disease categories (mental and behavioural disorder, epilepsy, dementia, depression and schizophrenia) are collected at secondary level of care; while only two conditions (“behavioural disorders” and “epilepsy”) are collected at the primary care level. In Nepal, mainly the morbidity and mortality data on a total of 67 disease categories (including depression, psychosis, anxiety, mental retardation, conversion disorder, alcoholism and self-harm/suicide) relating to mental health, based on ICD 10 categorization, are collected at the regional and national hospital level, while at the district and PHC level data on seven disease categories are collected. In Uganda, data on eight disease categories (anxiety disorder, mania, depression, schizophrenia, alcohol and drug use, epilepsy, childhood mental disorders and other forms of mental illness) are recorded.

In South Africa, mental health indicators relating to mental health case load, mental health visits and voluntary and involuntary admission rates of people below 18 years and older are recorded. South Africa and Uganda are the Emerald countries which have Child and Adolescent Mental Health Indicators in their current HMIS. In Ethiopia, the data are disaggregated into child/adult so information on child mental health is available.

The insufficiency of mental health indicators in existing HMIS in Ethiopia and South Africa has been recognized and efforts to include additional indicators are being made. As a result, the Ethiopian national mental health strategy has specified various mental health indicators to be included in HMIS. In South Africa a proposal to expand the list of available indicators is suggested in the National Mental Health Policy Framework and Strategic Plan 2013.

In the remaining countries no such initiatives have been taken yet. However, all countries are in the process of considering possible amendments of existing HMIS to include additional mental health indicators. The envisaged strategies and processes required to amend HMIS in the respective countries include: advocating for change with the planning and policy directorate (Ethiopia), conduct focus group discussions with stakeholders, make a list of prioritized indicators and submit them to the government (India), consult stakeholders and advocate for a new policy (Nepal), amend the mental health policy by legislation and engage with the Directors of Planning, Research and Statistics at the Federal and State Ministries of Health (Nigeria), engage national and provincial managers in initial adaptation of the mental health action plan and its endorsement by the Department of Health (South Africa), and hold regular review meetings and submit a request for amendments (Uganda).

The Emerald countries also face several challenges with regards to including additional mental health indicators in the HMIS, which include: concern from policy makers about indicator overload and competing priorities (Ethiopia), low priority of mental health and consequently a low availability of resources and skilled human resources (India and Nepal), the lack of political will for mental health reform and slow process of effecting changes (Nigeria), growing competition among several programs to incorporate additional indicators in HMIS (South Africa) and the length of the existing HMIS tool and lack of qualified staff at the health facility level (Uganda).

### Processes and mechanisms for data collection and management

While for other general health conditions the lowest level of HMIS data collection is the community, the primary health care centre and district level are the lowest level for mental health data. All six countries have standard HMIS formats for data collection. See Table 5 for further details.

**Table 5:** Data collection, compilation, reporting and dissemination

Themes	Ethiopia	India	Nepal	Nigeria	South Africa	Uganda
Data collection	Only paper and pencil	Paper pencil and electronic	Paper pencil and electronic	Paper pencil and electronic	Paper pencil and electronic	Paper pencil and electronic
Data compilation	Manually	HMIS web portal	HMIS online data entry system	Manually and electronically	Manually and electronically	Manually and electronically
Data analysis	Annually	Monthly	Quarterly and annually	Quarterly and annually	Quarterly	Quarterly
Frequency of data reporting to MoH	Quarterly	Monthly, quarterly and annually	Monthly	Monthly	Quarterly	Monthly, quarterly and annually
Data quality control mechanisms	Yes	Yes	Yes	Yes	Yes	Yes
Feedback mechanisms to the lowest level	Not clear	Yes	Yes	Not specified	Yes	Yes
Dissemination of HMIS data	Yes	Yes	Yes	Yes	Yes	Yes
Public access of HMIS report	Yes	Yes	Yes	No	Yes	Yes

Though electronic HMIS are being piloted in certain health facilities, Ethiopia largely uses paper forms, whereas the other five countries use both paper and electronic formats for data collection. In Nepal, India and South Africa, data from the district level upwards is compiled electronically, either through an online system or web portal.

The HMIS in all six countries are subject to systematic monitoring and evaluation, and data control mechanisms. All countries make checks for completeness, timeliness and validity of data. Different countries utilise different processes; Lot Quality Assurance Sampling (Ethiopia), systematic monitoring and evaluation (M&E) and 52 validation questions (India), review meetings, data verification meetings and field visits (Nepal), data review and verification meetings (Nigeria) and Standard Operation Procedures (South Africa) are used. Data verification meetings are the most commonly used method for data quality control but the frequency of the meetings varies across countries. Nepal has half yearly verification meetings while South Africa conducts data clean-up workshops monthly (at the health facility level) and quarterly (at the provincial and national level). In Nigeria, monthly data collation occurs at the district level while the verification exercises takes place (monthly) at the state level. The processes of data cross-checking also vary. India compares different indicators and analyses several interviews to cross-check the data, whereas in Uganda feedback is also given through “Barazas” (consumer and stakeholders group) meetings.

The countries also vary in their dissemination plans. India has a national dissemination plan and HMIS data are disseminated through periodic workshops conducted at different levels. In Ethiopia, Nepal and Nigeria data are disseminated annually in the form of public reports. In the case of Uganda, dissemination is done through quarterly review meetings at the ministry, district and lower health facility level. All countries have public access to government data. However, in Nepal, Nigeria and South Africa, this can be fully accessed following approval of a formal request.

## Discussion

The six Emerald countries face substantial contextual and health governance challenges in developing and implementing information systems that are able to adequately record, report, analyse and disseminate mental health information. Irrespective of income level (based on countries’ gross national income), similar challenges and opportunities are seen in developing and implementing HMIS. The Ugandan and South African mental health policies specifically mention HMIS. The Ethiopian mental health strategy also has provisions for mental health information collection and management, but it is not yet implemented. In other countries there is no specific explanation on how and from where mental health information should be collected. This cross-sectional situation analysis shows that there are no separate mental health information systems, but that some mental health indicators are collected through routine HMIS. In general, the countries tended to report the status of mental health (morbidity and mortality indicators per disease category) rather than system level indicators such as quality and utilization of services, average length of stay, bed occupancy rate, rates of admissions and social welfare benefits given to people living with mental health problems to cover treatment expenses. Notably, mental health referrals and mental health training data are scarcely collected.

Due to funding and government priorities, the HMIS in Emerald countries appear to be more geared to communicable diseases, overlooking the information management systems need for non-communicable diseases like mental health. With the epidemiological transition from communicable to non-communicable diseases (NCDs) the kind of indicators needed for mental health are also relevant for other NCDs so the political emphasis on NCDs may support the changes in mental health. The capacity to track changes in treatment coverage and quality of care is essential for monitoring the impact of mental health programs [22]. It is therefore important to install or strengthen the existing information systems that can appropriately inform the planning and implementation of mental health care. The inclusion of new indicators within the HMIS is not an easy task; several challenges need to be overcome. In order to develop functional mental health information sub-systems within HMIS, there is a strong need for lobbying and advocacy with stakeholders at the district, regional and ministry level, in order to convince policy makers to develop political will for mental health reform and to break the cycle of slow progress in effecting changes.

The findings indicate that there are procedures and mechanisms in place for data collection, compilation, reporting, analysis, feedback and dissemination, but due to low number of HMIS experts and HMIS staff trained on mental health, it is difficult to fully implement the procedures and mechanisms. The low number of HMIS experts in study countries (twenty in India, five in Nepal, three in Uganda and none in remaining countries) shows that countries depend upon junior level and in general non-qualified staff for the majority of health information management. This raises questions about timely supervision of junior staff and the quality of data generated by staff without much technical guidance. The lack of specialist HMIS-related courses in academic institutions and lack of political will of policy makers and planners within ministries of health might be some of the causes for the low number of HIMS experts in these countries.

There have been some positive developments in Ethiopia, and Uganda, however, where institutions have started providing specialized courses on HMIS. The lessons gleaned from these countries could be useful in advocating for specialized HMIS courses in other Emerald countries. Agreeing with Littlejohns, Wyatt and Garvican [23], who argued that educational efforts of HMIS staff often concentrate on how to use the system rather than why it should be used, we stress the important role of academic institutions in providing specialized courses on health informatics and thereby contributing to HMIS strengthening.

The findings of the study suggest that mental health data collected through routine HMIS in study countries is inadequate and does not reach policy makers on time to influence policies. This may be due to a lack of consensus about the information needed, between data producers and data users at each level of the health system [2]. This could be further linked with a lack of clear policy guidelines on mental health data collection and management. Secondly, due to the lengthy process of data collection, recording, reporting and analyzing, the findings do not reach decision makers in a timely way; hence decisions are often made without any information input [2].

In order for health information to influence policy making decisions, the data have to be of high quality and relevant for decision makers at each management level [2]. The decision makers, such as policy makers, planners and health service managers, at the district and national level require evidence based information to formulate policy and planning. It is debatable as to which data sources are preferable for developing and tracking health system targets. It has been argued that household and facility surveys yield better quality information than routine HMIS [5] because they are less biased and conducted by a dedicated team of more skilled researchers. Others perceive HMIS to be costly, producing low quality and irrelevant information [24], thereby contributing less to the decision-making process. We argue that, despite many challenges such as poor design and low capacity of health workers to manage information, HMIS do allow for routine tracking of progress towards organizational objectives and improving health system performance. We are of the view that the HMIS data are more timely and relevant to inform decision-making by managers of health services compared to population surveys that do little to inform the day- to- day management of health services. However, we acknowledge that HMIS is not the only data source relevant for decision makers. There are various other data sources, for example, causes of death obtained from civil registration which can provide suicide rates; while population surveys which can provide prevalence estimates for mental health problems. The study findings also suggest that countries do not see the alternative of HMIS rather they are in the process of developing mental health indicators within HMIS. We also believe that the current HMIS, if re-structured properly with adequate human resources, can yield reliable mental health information that is useful for improving service provision and policy making.

Introducing a separate mental health information system (MHIS) is unrealistic and undesirable in LMICs where mental health is still largely neglected and public sector mental health human resources are in short supply, even more so for HMIS staff trained in mental health. Also, due to inadequate government attention to mental health, a very limited budget is allocated which would be unlikely to sustain a separate/independent mental health information system. Secondly, a separate MHIS would be against the principle of integration of mental health into general health care. We argue therefore against a parallel MHIS and stress the importance of re-structuring of the current HMIS to include sufficient mental health indicators. The re-structuring should also include provision of infrastructure and software support and training of HMIS staff regarding mental health in general and mental health information management in particular.

The HMIS of six countries under study already collect mental health information, so we see that there is an opportunity to strengthen existing HMIS to obtain better quality mental health data. HMIS is not just introducing statistical techniques, it is “introducing a new management approach with wider organizational consequences” [25]. Therefore, it is important to re-structure not only the HMIS but also the health governance mechanisms and organizational management culture to get better mental health information that will be useful for service provision and policy making. In theory, many approaches to HMIS such as managerial, infrastructural and organizational exist in the literature [9], but in practice greater emphasis is placed on technical approaches of systematic data collection, ignoring the reality that HMIS goes beyond technical aspects and incorporates complex social, institutional and cognitive realities [9, 26].

### Limitations of the study

One of the limitations of the study is that it largely relied on secondary information available in the public domain. The study therefore might not have given a complete picture of all the available information. Efforts were made to validate the information by informally interviewing the responsible government officials. Secondly, the study gives the overall context of the HMIS and the mental health indicators within it but it does not assess the performance of HMIS. An assessment using an established assessment framework such as the performance of routine information system management (PRISM) as suggested by Aqil, Lippeveld and Hozumi [5] and the Health Matrix Network’s assessment tool might have been ideal. But, in the context of limited time and resources, this was not possible. Thirdly, in the absence of previously published reports, this cross-country situation analysis was unable to investigate the attitudinal aspects of policy makers, planners and health workers’ willingness to develop and implement mental health information sub-systems within HMIS. This will be addressed in another study, planned to evaluate the effectiveness of additional mental health indicators, integrated into the routine health management information system. Another limitation is that we have only considered HMIS for government health services. Thus, it does not provide information about the private sector which provides a significant proportion of health services in many LMICs. Nonetheless, this situation analysis provides data and contextual factors that have value for the development, implementation and evaluation of mental health information sub-system within routine HMIS.

### The future of mental health information systems in low and middle income countries

We propose the following five steps for developing and managing mental health information sub-systems that are integrated into routine HMIS. Firstly, there needs to be policy and management level clarity on organisational and system level changes that are required for the integration of mental health components into routine HMIS. We believe that the organizational change should be as part of the system development process, not merely as a technical innovation. Secondly, there is a need to examine information systems through information audits to identify factors that would facilitate or inhibit adoption of mental health information sub-systems within routine HMIS. The evaluation of HMIS should be multi-dimensional, covering many aspects beyond technical functionalities [23]. Thirdly, capacity building in HMIS human resources needs to be strengthened [25] and staff should be trained in mental health, to collect and report data correctly. Given the lack of HMIS specialists and trainers available in-country across the sites, the regular onsite supervision of junior staff is not realistic. An alternative could be the implementation of a training of trainers (ToT) program in mental health information sub-systems within HMIS. Support to the newly-trained trainers through distance supervision, using modern telecommunication needs to be considered. Fourthly, a data handling mechanism should be developed or strengthened at all levels of the health care system. Finally, a culture of information use at each level of health facilities needs to be encouraged and in that capacity senior level health managers and decision makers could play an exemplary role by using and encouraging the use of health information [27].

## Conclusions

The six countries are at different stages in the development of HMIS as well as in the collection and reporting of mental health indicators, but the challenges and opportunities are similar across countries. The workforce, infrastructure, and software-related challenges are common. Strong policy and strategic vision for mental health aspects of HMIS is lacking in all the countries. The current HMIS in some of the countries collects few mental health data and there is a need to add indicators related to service need, coverage and utilization. There are very limited HMIS experts available and therefore the bulk of the work is done by junior staff without expertise and experience.

## Abbreviations

EMERALD: Emerging Mental Health Systems in Low- and Middle-Income Countries
HIS: Health Information Systems
HIT: Health Information Technician
HSIS: Health Sector Information Systems
HMIS: Health Management Information Systems
LMICs: low and middle income Countries
mhGAP: Mental Health Gap Action Program
MNS: mental, neurological and substance abuse
NCDs: non-communicable diseases
SoPs: standard operating procedures
ToT: training of trainers
